# Tab-to-Busbar Interconnections in EV Battery Packs: An Introductory Review of Typical Welding Methods

**DOI:** 10.3390/mi17010002

**Published:** 2025-12-19

**Authors:** Sooyong Choi, Sooman Lim, Ali Shan, Jinkyu Lee, Tae Gwang Yun, Byungil Hwang

**Affiliations:** 1 Department of Intelligent Semiconductor Engineering, Chung-Ang University, Seoul 06974, Republic of Korea; 2 Graduate School of Flexible and Printable Electronics, LANL-JBNU Engineering Institute-Korea, Jeonbuk National University, Jeonju 54896, Republic of Korea; smlim@jbnu.ac.kr (S.L.);; 3 Department of Molecular Science and Technology, Ajou University, Suwon 16499, Republic of Korea; 4 School of Integrative Engineering, Chung-Ang University, Seoul 06974, Republic of Korea

**Keywords:** tab-to-busbar interconnections, electric vehicle battery packs, ultrasonic welding, laser beam welding, resistance welding

## Abstract

This paper reviews tab-to-busbar interconnections in lithium-ion battery packs, focusing on resistance welding (RW), laser beam welding (LBW), and ultrasonic welding (USW). The functional roles of tabs and busbars and typical material choices (Al-, Cu-, and Ni-plated Cu) are outlined. Subsequently, the processes are compared in terms of heat input, interfacial metallurgy, electrical resistance, mechanical robustness, and manufacturability. USW, as a solid-state method, suppresses porosity and limits Al-Cu intermetallic growth, but is sensitive to thickness, stack geometry, and tool wear. LBW enables high-speed, automated production with precise energy delivery, yet requires careful control to mitigate spatter, porosity, and brittle IMCs in dissimilar joints. RW remains cost-effective and flexible but can suffer from electrode wear and variability with highly conductive stacks. This review also summarizes the effect of the busbar material (Al versus Cu) and thickness on the connection resistance and temperature increase under a high current. No single process is universally superior, and the selection should match the stack-up, reliability targets, and production constraints. This paper aims to provide an overview of recent and conventional research trends for each welding method and to introduce selected non-traditional approaches, thereby presenting a range of viable options for future applications.

## 1. Introduction

The global impetus toward sustainable energy and reduced carbon emissions has triggered a paradigm shift in the automotive industry, establishing electromobility as the foremost alternative propulsion technology. This transition necessitates extensive scale-up in the mass production of electric vehicles, in which the lithium-ion battery pack functions as the central energy-storage unit [1,2]. These packs are complex assemblies that typically follow a pack-module-cell hierarchy. Their performance, safety, and lifespan critically depend on the integrity of thousands of electrical interconnections [3,4,5,6,7].

The manufacture of these joints, which commonly involve highly conductive materials such as Cu and Al in dissimilar configurations, is significantly challenging. The resulting connections must ensure high electrical conductivity and robust mechanical strength. Mechanical fastening without heating or melting offers a simple process route; however, it may face limitations in ensuring long-term stability of contact resistance and securing reliability under vibration and thermal-cycling environments. In contrast, welding-based joining forms a more continuous and integrated metallurgical bond, providing lower and more stable electrical resistance and superior mechanical reliability, and is therefore advantageous for high-current EV battery applications. Among the various joining technologies available, resistance welding (RW), laser beam welding (LBW), and ultrasonic welding (USW) have emerged as the most prominent and competing processes for interconnecting battery cells, tabs, and busbars.

RW, which leverages the Joule heating principle, generates localized heat at the faying interface by passing a large electrical current through the joint under applied pressure. RW is a well-established, high-speed, and cost-effective process [8,9]. However, the application of RW to battery interconnects is challenging. The low intrinsic electrical resistance of highly conductive materials such as Cu and Al requires significantly high currents to generate sufficient welding heat, which can accelerate electrode wear and result in process inconsistencies. Furthermore, RW is sensitive to surface conditions, and the applied pressure can deform fragile cell components [10].

LBW is a noncontact, fusion-based process that is highly regarded for its exceptional processing speed, high power density, and minimal heat-affected zone, which make it highly suitable for high-volume automated production [11,12,13]. However, the application of LBW is complicated by the high reflectivity of Cu and Al. Notably, its fusion-based nature renders it susceptible to defects such as porosity and hot cracking. The substantial differences in the physical and thermal properties of dissimilar joints (such as Cu-Al) lead to the formation of thick, brittle intermetallic phases, which can severely degrade the long-term mechanical and electrical reliability of the joint [14,15,16,17,18].

In contrast to the thermally driven processes, such as RW and LBW, USW is a solid-state joining technique. USW employs high-frequency mechanical vibrations under pressure to create a metallurgical bond without the macroscopic melting of the base materials. This fundamental advantage of USW prevents fusion-related defects (such as porosity) and, crucially, minimizes the formation of detrimental IMCs, making it a preferred method for joining thin foils and dissimilar material combinations [19,20]. However, USW is limited by its sensitivity to material thickness and cleanliness, constraints imposed by joint geometry, and process variability resulting from tool (sonotrode) wear [21,22,23,24].

Therefore, the optimal choice between these three dominant technologies is not straightforward and necessitates careful consideration of tradeoffs regarding specific applications, material stack-ups, and production requirements. Consequently, a comprehensive understanding of the distinct advantages, limitations, and underlying mechanisms of RW, LBW, and USW is essential to optimize the manufacture of reliable and safe EV battery packs.

**Figure 1:**
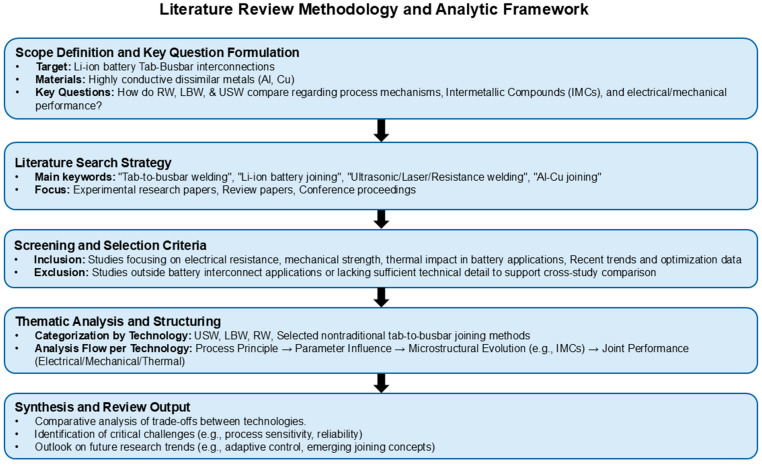
Literature review workflow and paper selection/categorization criteria for Li-ion battery tab-to-busbar joining.

Figure 1 provides an overview of the literature search, screening, and categorization workflow adopted in this review. The scope was defined around Li-ion battery tab–busbar interconnections involving highly conductive dissimilar metals (Al and Cu), with emphasis on elucidating process mechanisms, IMC formation/evolution, and resulting electrical and mechanical performance across RW, LBW, and USW. Literature was identified using targeted keywords (e.g., “tab-to-busbar welding,” “Li-ion battery joining,” “ultrasonic/laser/resistance welding,” and “Al-Cu joining”), focusing primarily on experimental journal articles, review papers, and relevant conference proceedings. Studies were included if they reported battery-relevant outcomes such as electrical resistance (including contact/interface resistance where specified), mechanical strength (with clearly defined test modes and failure characteristics), thermal impact under battery-applicable conditions and/or process optimization or trend data supported by explicit parameter windows. Studies were excluded when they addressed joining applications outside battery interconnects or lacked sufficient technical detail to enable rigorous cross-study comparison. The selected body of work was then analyzed using a consistent sequence, process principle, parameter effects, microstructural/IMC evolution, and joint performance (electrical/mechanical/thermal) to ensure a structured, technology-to-technology assessment of trade-offs, sensitivities, and reliability-relevant implications.

## 2. Fundamentals of Tabs and Busbars: Role, Importance, and Materials

The overall performance, safety, and longevity of high-voltage battery packs are fundamentally dependent on the quality of the numerous internal electrical connections [25,26,27,28,29]. The interface between the individual cell tabs and the busbar module represents a critical nexus. This section provides a foundational understanding of these core components, detailing their specific roles, the critical importance of their interconnections, and materials typically employed in their construction.

### 2.1. Role and Critical Importance of the Tab-Busbar Interface

The tab serves as the primary electrical terminal of a single cell, extending from the internal current collectors (anode and cathode foils) to the exterior of the cell casing. The tab is the conduit through which all energy enters and exits the cell [10,30,31]. The busbar is a larger conductive component designed to aggregate this energy by physically and electrically connecting multiple cell tabs, typically in series for voltage summation or in parallel for capacity augmentation [32,33,34,35,36]. Therefore, the integrity of tab-to-busbar joints is critically important. Suboptimal connections, characterized by a high electrical resistance or poor mechanical strength, can severely compromise the entire battery system. A high joint resistance leads to significant I^2^R (ohmic) losses, which manifests as waste heat and a reduction in the overall power output and energy efficiency of the battery pack. This localized heat generation can accelerate the degradation of nearby cells, and in extreme cases, poses a significant thermal runaway risk [37]. Furthermore, these joints must exhibit high mechanical robustness to withstand the shocks and vibrations inherent in applications such as electric vehicles. Failure at this interface results in an open circuit, rendering a segment of the battery module inoperable [38,39]. Consequently, the quality of this connection is not merely a manufacturing consideration, but a core engineering challenge. The selection of appropriate materials and robust joining methods are primary factors that directly govern the reliability and performance of an entire battery pack.

### 2.2. Tab and Busbar Materials

The performance of the tab-to-busbar interface begins with selecting appropriate materials that balance the electrical, thermal, mechanical, and economic requirements. In typical pouch-cell architectures, two external terminals or tabs project outward from the main cell body, penetrating the hermetic seals. The conventional material configuration designates Cu[Ni] as the negative terminal and Al as the positive terminal. Numerous studies investigated experimental frameworks based on this configuration and employed various thick Cu[Ni] and Al tabs [37,38,39,40,41,42,43,44].

A bus bar is required to connect the tabs to the broader battery module. The selection of the busbar material and its geometric thickness is a critical design decision dictated by essential operational parameters such as the overall current-carrying capacity of the module, its thermal management strategy, and the required electrical efficiency. The two predominant materials used for busbar applications are Cu and Al. Intrinsically, Cu possesses superior properties, including a lower electrical resistivity (higher conductivity) and higher tensile strength and thermal conductivity. However, this comparison is limited because it does not consider material density, a critical parameter in this analysis. The high density of Cu significantly affects the overall component mass. Normalizing the performance by weight reveals the advantages of Al. Aluminum offers a more favorable conductivity-to-weight ratio than Cu. This dichotomy in properties creates a distinct tradeoff. For weight-sensitive systems, such as automotive electric vehicle applications, where minimizing the overall mass is paramount, Al is often the preferred choice. Conversely, in scenarios where spatial constraints are the primary concern and component size must be minimized, the superior volumetric conductivity of Cu makes it a viable alternative [45,46,47]. Despite the clear importance of material choice, a comprehensive investigation into the effect of varying the busbar materials and thicknesses on the resulting joint quality from different welding methods is lacking in existing literature, highlighting the need for further comparative studies. Additionally, although surface characteristics play a critical role in material joining, only a limited number of studies have investigated these aspects in detail [48,49].

## 3. Joining Technologies for Tab-to-Busbar Connections

The joining technology employed to create a robust and reliable connection between the tab and the busbar is critical. In particular, Cu is often used as the material for tabs or busbars and Cu is one of the materials that oxidizes very easily. In joining or welding fields, much research has been conducted attempting to remove this oxide layer well [50,51,52,53,54,55]. Each welding process has its own characteristics, and the choice of welding method may also vary depending on the materials being used.

The choice of the welding method directly influences the final electrical, mechanical, and thermal properties of the joint, with RW, LBW, and USW emerging as the three dominant industrial processes owing to their precision and control.

### 3.1. USW

USW is a prominent solid-state joining solution for battery tab and busbar interconnections and offers a distinct alternative to fusion-based processes. This technique utilizes high-frequency mechanical vibrations coupled with clamping pressure to create a metallurgical bond at the atomic level, typically without requiring the macroscopic melting of the base materials. The primary motivation for its use, particularly with dissimilar materials such as Al and Cu, is its ability to minimize the formation of thick brittle IMC layers, which are common failure points in fusion welds [56,57,58,59,60]. Raj et al. [61] systematically investigated how key USW parameters (pressure, weld time, and amplitude) influence joint resistivity and interfacial microstructure in Al-Cu dissimilar wire joints using a design-of-experiments approach, complemented by optical and SEM analyses. Their results indicated that increasing ultrasonic energy led to lower joint resistance while concurrently reducing the extent of detrimental IMC formation, with higher-energy conditions producing more uniform weld patterns and fewer interfacial defects.

Das et al. [31] conducted a feasibility study on automotive battery tab interconnects, comparing ultrasonic metal welding, resistance spot welding, and pulsed TIG spot welding for similar and dissimilar Al and Cu[Ni] tab joints using lap shear and T-peel tests; they reported that lap shear strength was generally four to seven times higher than T-peel strength and proposed a lap shear-to-T-peel strength reduction ratio as an additional indicator for joining-process selection. In subsequent work from the same research group on multi-layer Al-Cu tab-to-busbar joints, three 0.3 mm Al tabs were ultrasonically welded to a 1.0 mm Cu busbar and the effects of amplitude, pressure, and time on lap shear and T-peel loads were quantified [21]. For this representative stack-up, the maximum lap shear load increased from ~1534 to 1905 N with increasing amplitude, while the maximum T-peel load rose from ~113 to 613 N, indicating that peel resistance can be particularly sensitive to ultrasonic energy input. Likewise, Silva et al. [22] evaluated ultrasonic spot welds between Cu and Al plates and, for a nominal overlap area of 9.0 mm^2^, reported average tensile-lap failure loads spanning approximately 587–948 N across their parameter window. These results collectively suggest that, although lap shear remains the higher-load metric for Al-Cu interconnects, peel performance should be concurrently considered when defining process windows and quality criteria for multi-layer tab-to-busbar configurations.

**Figure 2:**
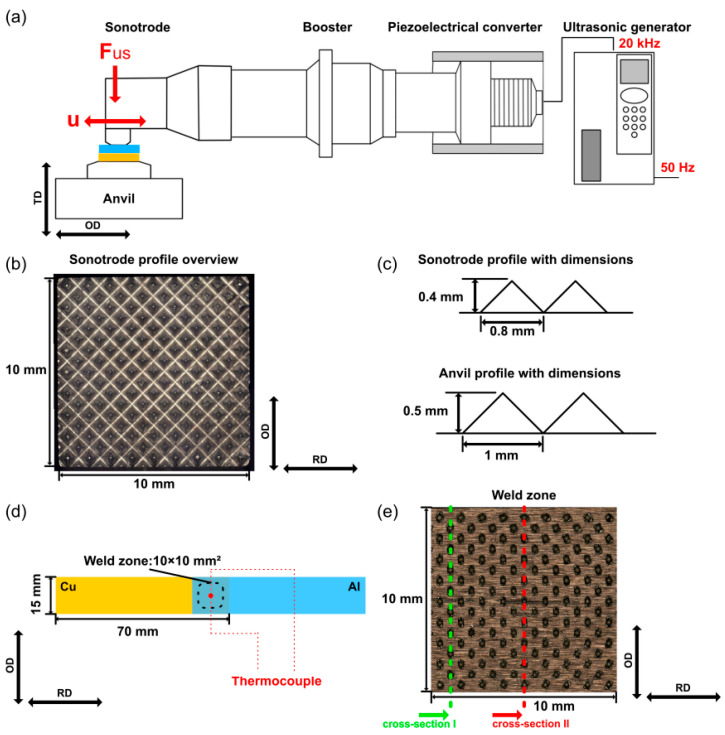
Experimental setup: (**a**) schematic of an ultrasonic welding system, (**b**) optical micrographs of sonotrode profile, (**c**) dimensions of sonotrode and anvil profiles, (**d**) configuration of the joining partners, and (**e**) cut positions of cross-sections [62].

Recent fundamental studies have provided deeper insight into the weld formation mechanism at the microstructural level. Li et al. [62] comprehensively evaluated Al-Cu ultrasonic welds and systematically correlated the welding time with plastic deformation, microstructural evolution, and mechanical properties. Utilizing the 20 kHz USW system detailed in Figure 2, their experimental configuration involved an overlap joint with specialized pyramidal profiles on the sonotrode and anvil to ensure effective vibration transmission. Their results revealed that plastic deformation was primarily concentrated on the softer Al side, leading to dynamic recrystallization and grain growth near the interface.

**Figure 3:**
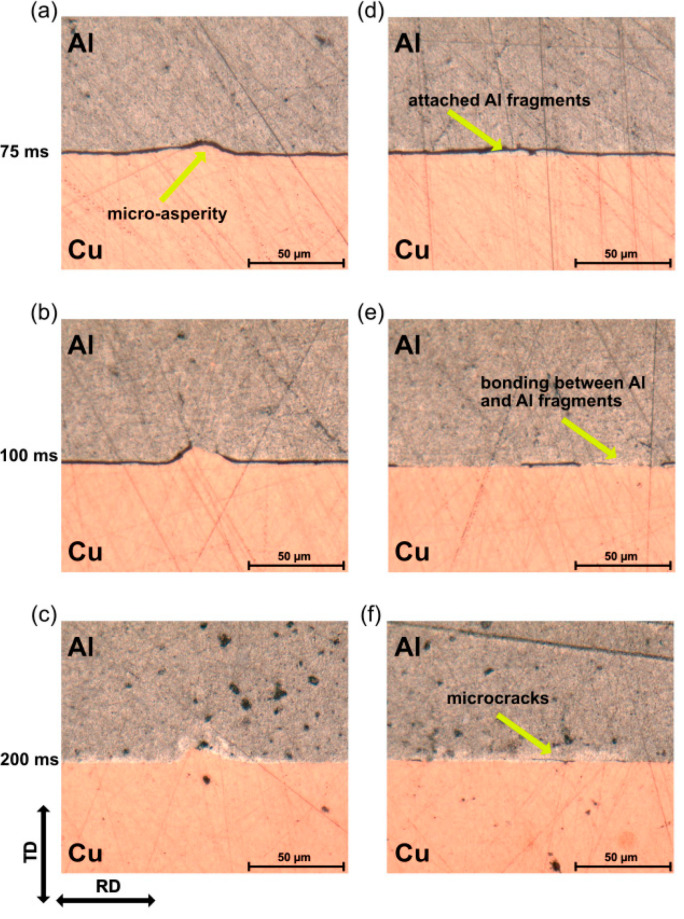
Optical micrographs of cross-sections at the weld interface: (**a**–**c**) microasperities; (**d**–**f**) locations with attached Al fragments [62].

This study proposed a complex weld-formation mechanism that begins with Al fragments attaching to the Cu surface, resulting from an initial bond-and-fracture event. Subsequently, a robust metallurgical bond forms between the bulk Al and these fragments. As shown in Figure 3, the microstructural development at the interface shows that weld formation initiates around the microasperities and Al fragments.

**Figure 4:**
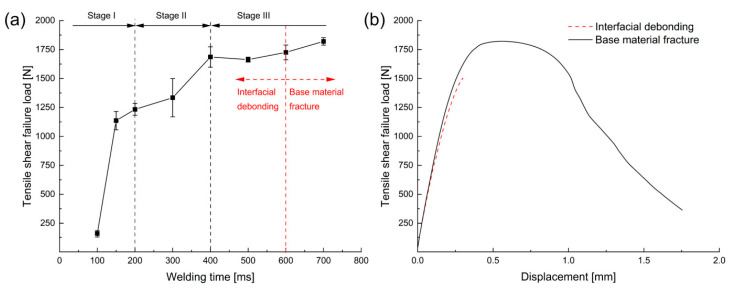
(**a**) Effect of welding time on the tensile shear failure load, and (**b**) load-displacement curves for interfacial debonding and base material fracture modes [62].

These local microwelds subsequently coalesce to form a continuous weld line as the welding time increases. This interfacial evolution is directly correlated with the mechanical properties of the joint. As shown in Figure 4, the tensile-shear failure load increases rapidly up to 400 ms before stabilizing. Notably, this figure also shows that the failure mode at welding times exceeding 600 ms transitions from brittle ‘interfacial debonding’ to robust ‘base material fracture,’ which exhibits significant ductile deformation. This detailed analysis provides fundamental guidance for process optimization by demonstrating that plastic deformation, microstructural changes such as DRX, and the expansion of the welded area are key factors in determining the final joint strength.

Electrical resistance is the principal metric for joint quality. Brand et al. [63] investigated interconnecting cylindrical cells and found a clear dependency between the weld area and resistance, with the resistance increasing with a decreasing joint area. They evaluated the resulting resistance to be acceptable and comparable to that of RW. Shin and de Leon [64] highlighted the challenges of multilayer joining, which is common in busbar configurations. They found that the presence of unbonded interfaces in multilayer Cu-Al stacks, which are potential USW defects, increased the connection resistance, although the values were still superior to those of traditional solder joints. The criticality of this metric led to its use in quality assurance, with McGovern et al. [65] measuring connection resistance as part of the QA methodology for various welding conditions. Furthermore, the long-term stability of this resistance is a concern. Zhao et al. [66] found that fatigue increases the connection resistance of ultrasonically welded Al-Cu samples.

**Figure 5:**
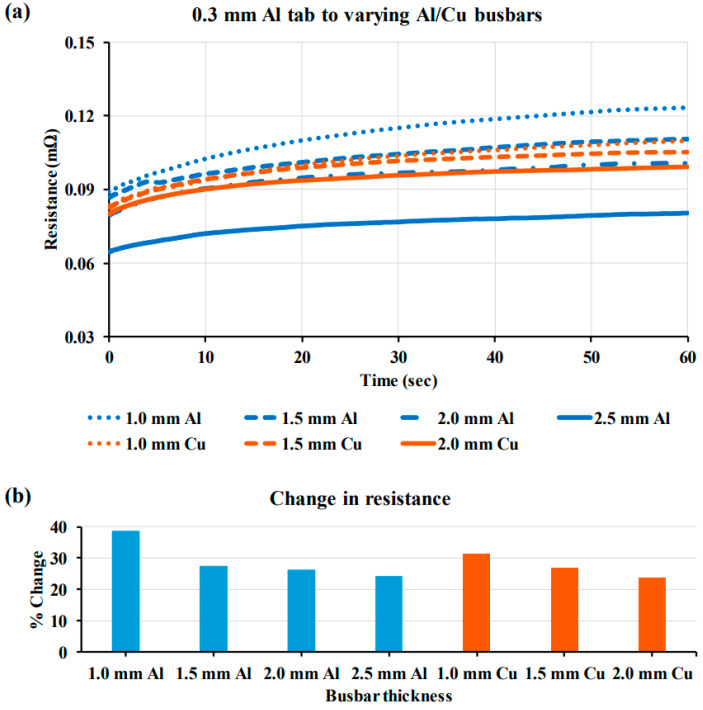
(**a**) Electrical resistance profiles for 0.3 mm Al tab to Al/Cu busbars; and (**b**) the percentage (%) changes in resistance [40].

**Figure 6:**
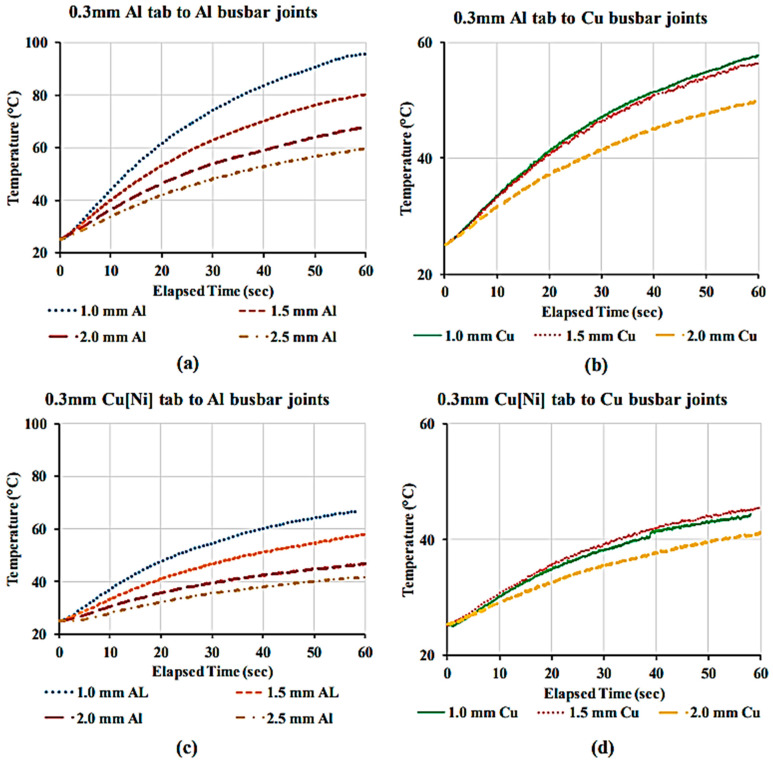
The temperature rise profiles due to application of 250 amp current for 60 s through (**a**) a 0.3 mm Al tab to different Al busbar joints; (**b**) a 0.3 mm Al tab to different Cu busbar joints; (**c**) a 0.3 mm Cu[Ni] tab to different Cu busbar joints; and (**d**) a 0.3 mm Cu[Ni] tab to different Cu busbar joints [40].

Das et al. [40] comparatively analyzed ultrasonically welded joints by systematically varying both the busbar material (Al and Cu) and thickness (1.0–2.5 mm). This study focused on quantifying the critical-to-quality criteria by establishing optimal welding parameters using T-peel tests to ensure a consistent level of mechanical strength for all test specimens. Subsequent high-current tests (Figure 5) revealed that increasing the thickness of both the Al and Cu busbars expectedly decreased the initial electrical resistance. Although the Cu busbars consistently exhibited a lower resistance than the Al busbars of the same thickness, this study highlighted the potential for interchangeability through thickness optimization. These electrical characteristics directly affect the thermal behavior, as illustrated in Figure 6. Joints with higher resistance generated more heat, with the Al tab to a 1.0 mm Al busbar combination showing the highest temperature increase, whereas the Cu[Ni] tab to Cu busbar joints exhibited the best thermal stability. This study provided quantitative data essential for balancing engineering trade-offs between lightweighting (using Al busbars) and thermal management in battery pack design.

Despite being a solid-state process, USW generates significant localized heat owing to friction. Das et al. [40] and Brand et al. [63] reported that USW exhibits higher heat generation than LBW or RW, although they evaluated the heat input to the battery itself as noncritical. However, in situ measurements challenge this assessment. Zhao et al. [66] and Li et al. [67] utilized thin-film sensors to measure temperatures as high as 660 °C near the weld zone, indicating substantial thermal loading. This heat generation directly affects operational performance. Das et al. [40] also investigated the electro-thermal behavior during current flow. They found that the connection resistance increases with joint temperature and that Al tabs resulted in higher heat generation than Cu tabs.

The mechanical stress induced during the USW process is its most significant drawback. Numerical studies by Kang et al. [68] and Lee et al. [69] revealed that the creation of a second weld on a single interconnector can induce sufficient vibrations to transmit stress and damage the existing weld joint. This complex interaction represents a critical manufacturing hurdle in ensuring the integrity of all connections within a module.

De Leon et al. [38] systematically studied the electrical resistance and thermal behavior of ultrasonic-welded tab-to-busbar joints for electric vehicle energy storage systems using a novel electro-thermo-mechanical evaluation technique. Their comprehensive analysis of various copper and aluminum configurations revealed that the applied tensile load was a critical factor significantly influencing the reduction of joint electrical resistance. This highlights the utility of the developed non-destructive evaluation system for precise optimization of lap configurations and welding parameters. This study further demonstrated the direct thermal consequences of environmental conditions on joint performance. As demonstrated in the low-temperature testing, the cooling of the interconnects considerably enhanced performance, with evaluations conducted down to cryogenic temperatures of 77 K showing marked improvements in conductivity. This performance boost was attributed to the minimized electrical resistance under specific tensile and thermal conditions, which were identified through the simultaneous monitoring system. These findings underscore that, although ultrasonic welding is a promising joining method, it must be rigorously evaluated under varying electro-thermo-mechanical loads to ensure long-term reliability and extend the life cycle of Li-ion battery cells.

These studies establish USW as a premier solid-state joining solution for EV battery interconnects, particularly for its ability to suppress brittle IMCs and optimize electrical-thermal trade-offs through parameter control. However, for USW to meet the rigorous demands of next-generation battery modules, several critical challenges must be addressed. The high-frequency mechanical vibrations inherent to the process pose a risk of fatigue or damage to adjacent welds, necessitating the development of advanced fixture designs and optimized welding sequences to mitigate stress propagation. Additionally, the challenges associated with unbonded interfaces in multilayer stacks and the potential for thermal damage to battery cells due to localized heat generation require the implementation of more sophisticated in-situ monitoring systems. Ultimately, as highlighted by the need for complex environmental testing, future research directions should shift towards establishing comprehensive reliability standards that rigorously evaluate joints under coupled electro-thermo-mechanical loads, rather than relying solely on static mechanical or electrical metrics, to ensure long-term performance under dynamic operational conditions.

### 3.2. LBW

LBW has emerged as a preferred technology for joining battery tabs and busbars because of its advantages as a high-speed noncontact process with localized energy input. The primary objective of LBW is to achieve a joint with minimal electrical resistance to ensure high efficiency and reduce ohmic heating. This joint quality is highly dependent on the process parameters.

**Figure 7:**
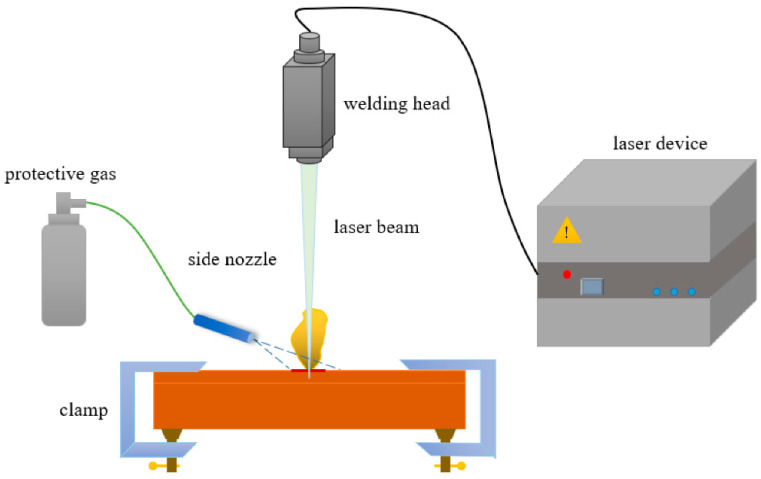
Schematic of laser welding [70].

The principle of LBW is simple. The laser welding process is conducted utilizing a high-energy laser beam to achieve material coalescence. As illustrated in Figure 7, the experimental setup typically comprises a laser device, a welding head, and a clamping fixture. The laser beam, often generated in a pulsed mode, is transmitted to the welding head where it is focused onto the joint interface of the lapped workpieces (e.g., ultra-thin plates). Concurrently, a protective gas such as argon is supplied via a side nozzle to shield the molten pool from oxidation during the thermal cycle.

Liebl et al. [71] investigated laser welding of pure copper using multi-mode fiber lasers at near infrared wavelength (λ ≈ 1 μm) to overcome the low absorptivity and process instability that currently limit the applicability of LBW to copper conductors in electrified powertrain and energy systems, systematically varying laser power (3–8 kW) and welding speed to delineate a process boundary between defect-free deep-penetration welds and regimes with melt ejections and spatter, employing cross-sectional analysis and high-speed imaging to attribute melt ejection to the formation and collapse of vapor bubbles in a bent keyhole, and, through regression-based modelling of penetration depth and seam cross-section as functions of process parameters, demonstrating that increasing laser power beyond 4 kW alters the trend of the process limit due to the growth of the molten pool volume, while additional experiments with helium shielding gas revealed that He shifts the process window toward lower welding speeds and suppresses melt ejections, concurrently transforming the weld pool geometry into wider and shallower beads with reduced cross-sectional area (on average ≈15% lower penetration depth, ≈25% larger bead width, and ≈20% smaller area compared to welding without shielding gas), an effect they interpret in terms of modified surface-tension-driven convection and enhanced convective heat dissipation at the pool surface, thereby establishing a physically grounded process window for stable, defect-minimized LBW of copper with industrial near-infrared multi-mode fiber laser sources.

Brand et al. [63] identified direct relationships between the weld seam configuration. In particular, its size, area, shape, and the resulting connection resistance.

Although electrical performance is critical, controlling the thermal input is a critical challenge. The high-power density of LBW, which is necessary to overcome the high reflectivity of materials such as Cu and Al, must be carefully managed. Brand et al. [63] evaluated the heat input using optimized parameters and reported it to be noncritical. However, excessive energy input or uncontrolled penetration can cause thermal damage to the heat-sensitive cell chemistry located directly beneath the casing. To mitigate this risk, strategies such as spatial power modulation (that is, beam wobbling or rotation) are recommended to control the penetration depth precisely [72]. Similarly, Schmitz [73] recommended a pulsed welding strategy with discrete seams to minimize the overall heat input.

The metallurgical challenges are equally significant, particularly those encountered when joining dissimilar materials, such as Cu-to-Al, which is common in battery modules. The fusion of these materials can lead to the formation of brittle IMCs, which negatively affect both the mechanical strength and electrical conductivity. This aligns with the findings of Shaikh et al. [74], who investigated the LBW of Cu to Ni-plated steel. They suggested that the metallurgical behavior was responsible for the observed increase in resistance, which was accompanied by an increase in joint strength. However, the precise relationship between the joint geometry and electrical properties remains complex. For example, Schmitz [73] found no direct correlation between the total joint area and electrical resistance, contradicting other reported findings.

**Figure 8:**
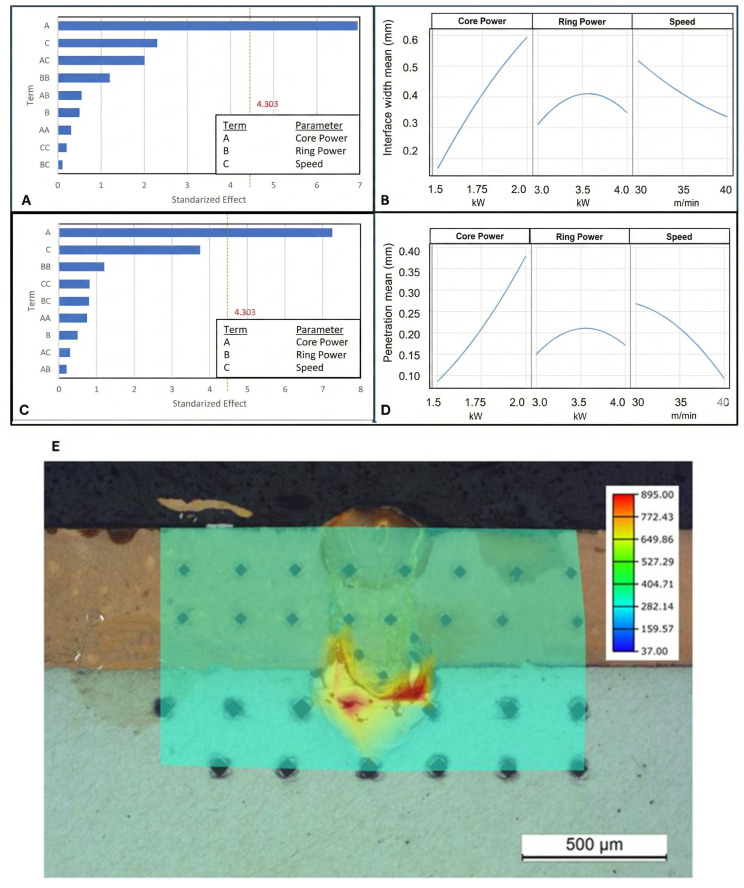
Standardized Pareto chart (**A**) and parameter response for interface width (**B**). Standardized Pareto chart (**C**) and parameter response for penetration (**D**) of Al-Cu joints. (**E**) Microhardness map of sample [44].

**Figure 9:**
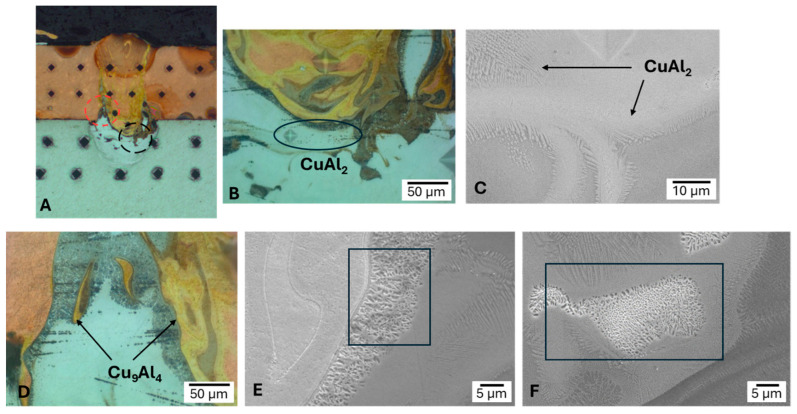
Optical and SEM images of sample 2 showing the microstructures generated. In (**A**), the macrograph indicates the area from which images (**B**,**C**) were taken, indicated by a black circle, and the area corresponding to images (**D**–**F**) is marked with a red circle [44].

Taboada et al. [44] systematically studied the effects of welding parameters on similar (Al-Al) and dissimilar (Al-Cu) joints for tab-busbar interconnects using advanced dual-beam and beam-shaping techniques. Their statistical analysis of Al-Cu joints, summarized in the Pareto chart in Figure 8A, revealed that the core power was the most statistically significant factor influencing both the penetration depth and interface width. This highlights the critical need for precise power control to manage the heat input and weld geometry. This study further demonstrated the direct metallurgical consequences of this process. As shown in the microhardness map in Figure 8E, the formation of IMCs at the Al-Cu interface considerably increased hardness, reaching a peak value of approximately 900 HV. This extreme hardness was attributed to the formation of complex and brittle IMCs, such as Cu_9_Al_4_ and CuAl_2_, which were identified through microstructural analysis, as shown in Figure 9. These findings underscore that, although laser parameters can be optimized for penetration, they must also be carefully balanced to minimize the formation of these detrimental phases.

Advanced laser strategies have been explored to address the combined challenges of process instability (such as spatter) and detrimental IMC formation. Pantsar et al. [75] used pulsed green lasers or nanosecond-pulsed infrared lasers to improve process stability, suppress spatter, and limit IMC growth, thereby enhancing both weld strength and conductivity. Other approaches target the material surface. Helm et al. [13] used ultrashort-pulse laser radiation to create surface structures that reduce the reflectance of Cu, thereby enabling a faster transition to deep penetration welding. Given this process sensitivity, the imperative of ensuring quality has necessitated the development of closed-loop in-process quality control methods such as those developed by Franciosa et al. [76] for battery assembly lines.

Building upon their initial investigation which established the correlation between wobble amplitude, weld geometry, and static strength, Asirvatham et al. [77] expanded their research to address the critical question of long-term dynamic reliability in Al-Steel joints. Moving beyond the geometric optimization of their previous work [78], this subsequent study identified the interaction time, controlled by the wobble amplitude and traverse speed, as the defining factor influencing energy distribution. This refined analysis highlights the critical need for precise parameter control to maintain short interaction times (<25 µs) and large inter-wobble distances (>150 µm). This study further demonstrated the direct metallurgical consequences of this process, providing a deeper insight than the initial findings. As evidenced by the microstructural evolution, optimizing the process successfully suppressed the formation of detrimental, crack-prone Al-rich Fe_2_Al_5_ phases, favoring instead the formation of Fe-rich IMCs and Al-Fe_4_Al_13_ eutectic phases. Most significantly, these metallurgical improvements translated into excellent fatigue performance, with joints withstanding 1 million cycles at 175 N. These findings validate the viability of using lighter, cost-effective aluminium busbars in battery interconnect applications, confirming the durability that remained a question in their earlier static analysis.

Research attempting improvements through material changes rather than process changes is also actively underway. Michele et al. [79] investigated the specific influence of nickel plating thickness on copper-to-steel interconnects using both single-mode and beam-shaping fiber lasers. Their statistical characterization revealed that the plating thickness is a decisive factor governing the weld bead interface morphology, particularly when utilizing a single-mode source. In contrast, for the beam-shaping source, the laser fluence was identified as the primary driver for penetration depth and interface width. These findings highlight that robust process optimization requires not only the control of laser parameters but also a strict accounting for the variability in surface coating thickness to guarantee consistent joint quality.

These findings highlight that robust process optimization requires not only the control of laser parameters but also a strict accounting for the variability in surface coating thickness to guarantee consistent joint quality.

While Michele et al. [79] highlighted the sensitivity of the process to surface coatings, recent research has sought to develop methods with higher process tolerance to overcome these limitations. Sun et al. [80] introduced a remote laser welding approach for joining thin copper wires to copper busbars in a T-joint configuration, specifically designed for the rapid assembly of electric motor windings.

A distinguishing feature of this method is the utilization of the wire itself as a filler material. This innovative approach ensures effective gap filling between the wire and the busbar, achieving a robust electrical and mechanical connection throughout the entire busbar thickness. Unlike previous findings that necessitated strict surface control, Sun et al. demonstrated that this approach exhibits high tolerance against varied pre-welding surface conditions. The joints maintained comparable mechanical, electrical, and thermal performance regardless of the surface treatment applied to the copper busbar. Furthermore, the process achieved a remarkable mechanical load capacity of 221 N, equivalent to 92.5% of the strength of the original enamelled wire. This distinct capability to maintain high performance without rigorous pre-welding surface preparation marks a significant step forward in the practical applicability of LBW for mass production.

Complementing these experimental process optimizations, Kumar et al. [81] focused on the structural validation of laser-welded joints, specifically for electric vehicle (EV) crash safety. In their study on busbar-to-terminal interconnects, they performed overlap welding on AA1050 aluminum sheets using a 1 kW continuous wave (CW) fiber laser integrated with a wobble head. The welding process utilized a specific rectangular weld pattern to ensure joint integrity.

To analyze these joints, the researchers addressed the computational inefficiency of traditional fusion-based modeling. Instead of simulating the complex physics of the molten pool, they developed a simplified structural model using LS-DYNA, tuned to experimental data from the rectangular wobble welds (including lap shear, T-peel, and torsion tests). This approach was successfully extended to predict the strength of 1.5 mm 1060 Al busbars welded to 4 mm terminals. The model’s predicted load–displacement curves closely matched the test data, demonstrating that experimentally valid laser welding parameters can be effectively translated into computationally efficient models for full-vehicle crash simulations.

Expanding beyond standard electrical interconnects, Jia et al. [82] reviewed the capabilities of ultrafast (femtosecond/picosecond) laser welding for joining transparent materials to metals, a critical technology for integrating advanced sensors into battery systems. Unlike conventional methods relying on linear absorption, this approach utilizes non-linear absorption and localized heat accumulation to achieve precise bonding with minimal thermal damage.

The authors emphasize that advanced temporal-spatial beam shaping, such as Bessel beams and burst modes, is essential to overcome the challenges posed by the mismatch in thermal expansion coefficients between dissimilar materials. By enabling high-strength, hermetic seals without auxiliary interlayers, this technology offers a robust solution for passive, high-stability packaging of optoelectronic components within the battery module.

The current state of research on LBW for battery interconnects demonstrates its significant potential as a high-speed, precision joining technology. As evidenced by the reviewed literature, the primary advantage of LBW lies in its ability to achieve low electrical resistance and high mechanical strength through localized energy input. However, the process is governed by a complex interplay of parameters. Key findings indicate that while optimizing laser power and beam modulation (e.g., wobbling) is essential for controlling penetration depth and minimizing thermal damage to battery cells, the metallurgical compatibility of dissimilar materials remains a critical hurdle. The formation of brittle intermetallic compounds (IMCs), particularly in Al-Cu joints, presents a dual challenge: while some interaction is necessary for joint formation, excessive IMC growth leads to increased hardness and potential failure.

Despite advancements in beam shaping, pulse strategies, and tolerance-improving designs like wire-based filling, significant challenges persist regarding process sensitivity to surface conditions such as coating thickness. To address these limitations, future research must converge on several key areas. First, there is a critical need for the development of adaptive process control systems capable of real-time, closed-loop feedback to adjust laser parameters on-the-fly, compensating for variations in surface quality and gap fit-up. Simultaneously, research should explore advanced material solutions, including novel interlayers or surface treatments, that can suppress detrimental IMC phases in dissimilar metal joints without compromising electrical conductivity. Finally, these physical process improvements must be supported by holistic simulation approaches that bridge the gap between the computational fluid dynamics (CFD) of the melt pool and structural finite element analysis (FEA). Such integrated models are essential for accurately predicting long-term fatigue and electrical aging under the dynamic loads experienced by electric vehicles.

### 3.3. RW

RW is a well-established thermoelectric process widely applied in battery connections. The versatility of RW allows it to be adapted to various cell formats, including prismatic, pouch, and cylindrical configurations. The process generates heat at the workpiece interface via a high-amperage current governed by Joule heating (I^2^R), while the electrodes apply pressure to forge the components together. The high speed and cost-effectiveness of RW make it a common choice for battery-manufacturing applications.

**Figure 10:**
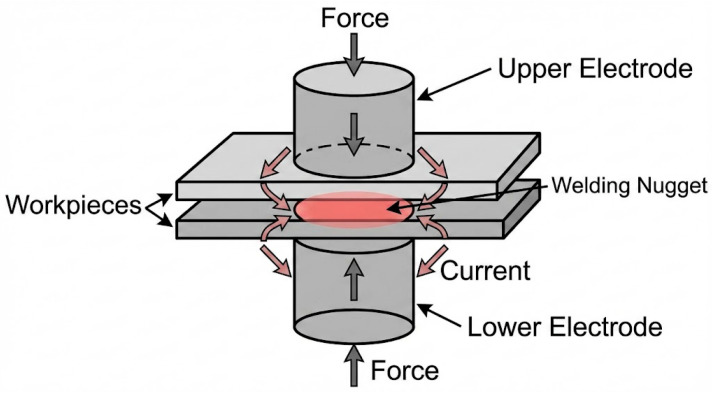
Schematic diagram of the resistance welding process.

Figure 10 illustrates the fundamental configuration of resistance welding. The process involves placing overlapping workpieces between an upper electrode and a lower electrode. A compressive force is applied through the electrodes to ensure intimate contact between the sheets, followed by the application of a high electric current. The electrical resistance at the faying interface generates localized heat, causing the material to melt and form a welding nugget. Upon cessation of the current, the nugget solidifies under sustained electrode pressure, creating a permanent metallurgical bond between the workpieces.

The quality of the resulting joint is highly sensitive to energy input. Godek [83] reported that increasing the energy input increased the weld diameter and correspondingly decreased the connection resistance. Similarly, the number of weld points is a critical factor. Brand et al. [63] demonstrated that increasing the number of weld spots progressively lowers the overall connection resistance, creating a more effective electrical pathway. They also noted that single-sided RW is particularly effective for joining materials with low electrical conductivity.

However, electrode degradation, which can lead to an inconsistent weld quality over time, is a significant operational challenge in RW. Brand et al. [63] reported that RW results in a considerably lower heat input than LBW or USW; however, the thermal impact remains a crucial consideration. The weld performance during battery operation is equally important. Liu et al. [84] investigated 18,650 cells with spot-welded interconnectors, and found that these joints contributed to increased heat generation during high-rate discharge cycles. This additional heat can impair the overall performance and health of the cell, demonstrating that the impact of welding persists beyond the initial manufacturing stage.

Expanding the scope to emerging manufacturing technologies, Bourgeois et al. [85] investigated the feasibility and reliability of resistance spot welding on additively manufactured (AM) aluminum and stainless steel battery tabs. Distinct from traditional approaches that rely heavily on destructive testing, this study utilized a microscopic resolution ultrasonic imaging system to non-destructively characterize the weld nugget interface and assess joint integrity. The research highlighted that while standard automotive welding parameters could be successfully applied to AM components, the surface roughness of the printed parts significantly influenced weld formation; specifically, reducing surface roughness through polishing resulted in larger effective weld nugget diameters compared to as-printed surfaces. Furthermore, the authors observed that unlike the well-defined circular nuggets typical of wrought alloys, welds in AM materials exhibited irregular, oval-shaped morphologies and increased porosity, demonstrating that the integration of additively manufactured components into battery systems requires tailored inspection protocols such as advanced ultrasonic imaging to ensure structural and electrical reliability.

Despite these advancements, RW faces distinct limitations compared to LBW and USW, particularly in the context of modern battery pack assembly. The process inherently struggles with the consistent joining of highly conductive and dissimilar materials often utilized in tab-to-busbar connections, where the requisite high currents can induce excessive thermal stress and accelerate electrode wear. Consequently, while LBW and USW have witnessed rapid adoption due to their precision and lower thermal impact, RW requires further comprehensive investigation to expand its process window for high-capacity applications. Future research directions must prioritize the development of advanced process control algorithms and real-time monitoring technologies to mitigate defects, alongside the optimization of electrode geometries to accommodate the evolving material architectures of next-generation battery systems.

### 3.4. Non-Conventional Joining Technologies for Tab–Busbar Interfaces

In the semiconductor field, intensive research has been devoted to micro-scale, fine-pitch bonding technologies for three-dimensional interconnection, such as Cu-to-Cu bonding. As demand for extremely fine bonding increases, various bonding methods have been researched beyond traditional joining and welding techniques. For example, Various bonding schemes, including chemical pretreatment [86,87,88,89] and thermocompression bonding [90,91,92], have been developed. Similarly, research into new welding methods beyond traditional approaches is underway for battery tab and busbar welding. However, bonding processes in EV battery packs are highly sensitive to temperature, which hinders the direct implementation of these semiconductor-oriented approaches. Nevertheless, alternative bonding techniques for EV battery tabs and busbars, beyond conventional joining technologies such as RW, LBW, and USW, have been actively investigated [93,94,95].

Mypati et al. [93] investigated an alternative joining route for EV battery tabs by employing friction stir welding (FSW) instead of conventional technologies such as RW, LBW, and USW, focusing on Cu–Al micro-thickness joints representative of cell tabs in HEV battery packs. Using FSW to lap-weld 0.3 mm Cu and 0.2 mm Al sheets, they demonstrated that an optimized parameter set yields joints with electrical conductivity only about 9% lower than that of base Cu, while maintaining a relatively uniform interface microstructure with Cu-rich intermetallic compounds such as Al_4_Cu_9_ and AlCu_4_, which contribute to both charge transport and mechanical stiffness. In addition, through potentiodynamic polarization and electrochemical impedance spectroscopy in a LiPF_6_ electrolyte, the authors showed that FSW joints exhibit significantly enhanced corrosion resistance compared with the base metals, and that appropriate control of tool rotational speed suppresses Kirkendall void formation, which refers to voids that form due to unequal interdiffusion rates between the two metals, while promoting the formation of Cu-rich IMCs beneficial to electrical conductivity and corrosion behaviour, thereby demonstrating the feasibility of FSW as a viable joining method for cell-to-cell and potentially cell-to-busbar connections in EV battery packs.

Zhang et al. [94] investigated an alternative joining approach for lithium-ion battery tabs by employing a solder-reinforced adhesive (SRA) process using Ni-coated Cu foils representative of pouch-cell tabs joined with eutectic SnPb solder balls embedded in an epoxy structural adhesive layer, while systematically varying the presence of flux and the adhesive area. Using this SRA-based joining method, they demonstrated that flux-coated solder balls markedly improve wettability and promote the formation of a continuous Ni_3_Sn_4_ interdiffusion layer at the solder–substrate interface, thereby eliminating interfacial cracks and shifting the failure mode from interfacial to cohesive, and that, for appropriately designed adhesive areas, the resulting joints exhibit lap-shear and coach-peel strengths and energy absorption that surpass not only pure adhesive joints but also USW joints (e.g., up to ~159% and ~769% higher than USW in lap-shear peak load and energy absorption, respectively), while achieving electrical resistances in the range of 0.05–1.53 µΩ, approximately 44% lower than comparable USW specimens, thus establishing SRA as a mechanically robust and low-resistance joining technology for battery tab applications.

Taken together, these studies demonstrate that non-conventional joining technologies such as FSW and SRA can provide tab and tab–busbar joints with electrical performance comparable to, or in some cases superior to, that of conventional RW, LBW, and USW, while simultaneously mitigating certain limitations associated with fusion-based welding of micro-thickness Cu–Al stacks. Nonetheless, several critical issues remain to be addressed before these methods can be widely deployed in EV battery packs. For FSW, the process window must be further refined to control heat input and tool–workpiece interaction so as to limit excessive growth of brittle intermetallic layers, prevent distortion or tearing of thin foils, and ensure stable weld quality in more realistic geometries, including multi-layer tab stacks and true tab–busbar configurations under constrained access. For SRA, the long-term thermo-mechanical and electrochemical stability of the adhesive matrix and intermetallic interfaces under automotive operating conditions (thermal cycling, vibration, humidity, and possible electrolyte exposure) requires systematic evaluation, and the scalability of precise solder/adhesive dispensing and curing in high-throughput manufacturing lines remains an open question, particularly with respect to repairability and recyclability. More broadly, future work should couple detailed electro–thermo–mechanical modeling with accelerated life testing under representative load profiles to establish standardized metrics for comparing these non-conventional processes with RW, LBW, and USW at the module and pack levels, and should explore hybrid process routes (e.g., combining low-energy solid-state joining with localized reinforcement or sealing) tailored to emerging cell formats such as pouch CTP, large prismatic cells, and tabless cylindrical architectures.

## 4. Conclusions

The performance, safety, and lifespan of EV battery packs are critically dependent on the quality of the numerous internal tab-to-busbar electrical interconnections. Manufacturing these joints is significantly challenging, particularly when joining highly conductive and often dissimilar materials, such as Cu and Al [96,97,98,99,100,101]. RW, LBW, and USW have emerged as the dominant and competing joining technologies. As summarized in Table 1, each process possesses distinct advantages and inherent limitations, making optimal selection a complex tradeoff.

Although RW is cost-effective and rapid, it suffers from process instability and rapid electrode wear when applied to highly conductive materials. LBW, a noncontact, high-speed process, is highly amenable to automation. However, it is challenged by the high reflectivity of Cu and Al. Crucially, its fusion-based nature promotes the formation of brittle IMCs in dissimilar joints, which compromises the long-term mechanical and electrical reliability. Conversely, as a solid-state process, USW effectively suppresses the formation of detrimental IMCs, offering a significant advantage in joining dissimilar materials. However, USW is limited by the induction of mechanical stress, tool (sonotrode) wear, and high sensitivity to material thickness and surface cleanliness.

From an electrical-resistance perspective, all three welding technologies can achieve contact resistances suitable for high-current EV operation, but they do so through different mechanisms and with different sensitivities. USW typically provides low and stable resistance when a sufficiently large and continuous weld area is formed, and it maintains favorable electro-thermal behavior even in dissimilar Al-Cu joints, provided that unbonded interfaces in multilayer stacks are minimized. LBW can deliver very low resistance through carefully designed seam geometries and controlled penetration depth, yet its fusion-based nature makes the electrical performance highly sensitive to IMC thickness, porosity, and spatter. RW, in contrast, reduces resistance primarily by enlarging nugget size and increasing the number of weld spots, but must contend with variability introduced by electrode wear, surface conditions, and the high currents required for Cu and Al. Thus, when joint resistance and its long-term stability are the primary constraints, solid-state approaches such as USW or optimized LBW of compatible material pairs tend to be favored over conventional RW.

The mechanical robustness of tab-to-busbar joints is equally decisive for pack-level reliability. Under optimized conditions, USW can transition from interfacial debonding to base-material fracture, demonstrating that very high shear and peel strengths are achievable, though the same high-frequency vibrations can transmit parasitic stresses to neighboring welds and fragile cell components. LBW offers a wide design space in terms of joint geometry (e.g., wobble patterns, multi-beam strategies) and can attain high static strength and excellent fatigue performance when metallurgical compatibility is managed and crack-prone IMCs are suppressed. RW remains attractive for its inherently forged joint structure and short cycle time, but local indentation, sheet deformation, and nonuniform nugget formation can degrade mechanical integrity—particularly in thin foils, additively manufactured tabs, and complex stack-ups. Consequently, the preferred process for a specific application often reflects whether fatigue life, peel resistance, or tolerance to vibration and crash loads is most critical.

Considering manufacturing efficiency and cost, RW provides the lowest barrier to entry, with mature equipment, short weld times, and relatively low capital expenditure, making it appealing for cost-sensitive or low- to mid-volume production. USW generally entails higher tooling costs and periodic sonotrode maintenance, but offers fast cycle times, good energy efficiency, and strong compatibility with multi-layer foil stacks, which can reduce the number of discrete joining operations at the module level. LBW requires the highest initial investment because of laser sources, scanning optics, and safety infrastructure; however, it enables the highest degree of automation, remote welding, flexible joint programming, and integration into inline quality monitoring systems. When amortized over large-scale EV production, LBW can therefore become highly cost-effective on a per-joint basis, particularly in architectures favoring prismatic cells, busbar circuits, and integrated sensor packaging.

The choice of busbar material and thickness further couples into these process-level trade-offs. Increasing busbar thickness and utilizing Cu instead of Al reliably decrease connection resistance and suppress temperature rise under high current, yet this must be balanced against weight targets and packaging constraints. In weight-sensitive automotive applications, Al busbars joined by USW or carefully controlled LBW can offer acceptable electrical and mechanical performance while enabling significant mass reduction, whereas Cu busbars joined by LBW or RW are advantageous in compact layouts where volumetric conductivity and thermal spreading dominate. A system-level optimization that accounts for conductor geometry, thermal management strategy, and crashworthiness is therefore indispensable.

Finally, emerging non-conventional joining routes such as friction stir welding and solder-reinforced adhesives have demonstrated that tab and tab–busbar joints can achieve electrical and mechanical performance comparable to, or better than, that of RW, LBW, and USW in specific use cases. Nonetheless, issues related to process scalability, long-term electro-thermo-mechanical stability, and recyclability under realistic EV duty cycles remain unresolved. Future research should therefore be pursued. Integrated electro-thermo-mechanical–fatigue models that link microstructure, contact resistance, and lifetime and adaptive, closed-loop process control for RW, LBW and USW based on real-time sensing. And comparative module- and pack-level benchmarking includes cost, throughput, and sustainability metrics. Such a holistic framework will be essential for selecting and tailoring joining technologies that simultaneously meet the increasingly stringent performance, safety, cost, and environmental requirements of next-generation EV battery packs.

## Figures and Tables

**Table 1:** Comparison of welding technologies.

Process Technology	Advantages	Disadvantages
RW	• Well-established process • High-speed • Cost-effective	• Difficult to weld high-conductivity materials (Cu, Al) • High electrode wear rate and process inconsistencies • Sensitive to surface conditions • Risk of component deformation
lbw	• Non-contact • Exceptionally high process speed • Precise with minimal heat-affected zone • Highly suitable for high-volume automated production	• High reflectivity of Cu and Al surfaces • Susceptible to fusion-based defects (e.g., porosity, hot cracking) • Forms thick, brittle IMC layers in dissimilar joints, degrading reliability
USW	• Solid-state process (no macroscopic melting) • Excellent for dissimilar materials (minimizes IMCs) • Effective for joining thin foils and multi-layer stacks	• Sensitive to material thickness and surface cleanliness • Process variability due to tool (sonotrode) wear • Mechanical vibrations can transmit stress to sensitive cell components

## Data Availability

No new data were created or analyzed in this study.

## Abbreviations

The following abbreviations are used in this manuscript:

EV	Electric Vehicle
RW	Resistance Welding
LBW	Laser Beam Welding
USW	Ultrasonic Welding
IMC	Intermetallic Compound
DRX	Dynamic Recrystallization
QA	Quality assurance
